# A stochastic vision-based model inspired by zebrafish collective behaviour in heterogeneous environments

**DOI:** 10.1098/rsos.150473

**Published:** 2016-01-13

**Authors:** Bertrand Collignon, Axel Séguret, José Halloy

**Affiliations:** Université Paris Diderot, Sorbonne Paris Cité, LIED, UMR 8236, 75013 Paris, France

**Keywords:** collective motion, agent-based model, zebrafish, visual perception, solid angle

## Abstract

Collective motion is one of the most ubiquitous behaviours displayed by social organisms and has led to the development of numerous models. Recent advances in the understanding of sensory system and information processing by animals impels one to revise classical assumptions made in decisional algorithms. In this context, we present a model describing the three-dimensional visual sensory system of fish that adjust their trajectory according to their perception field. Furthermore, we introduce a stochastic process based on a probability distribution function to move in targeted directions rather than on a summation of influential vectors as is classically assumed by most models. In parallel, we present experimental results of zebrafish (alone or in group of 10) swimming in both homogeneous and heterogeneous environments. We use these experimental data to set the parameter values of our model and show that this perception-based approach can simulate the collective motion of species showing cohesive behaviour in heterogeneous environments. Finally, we discuss the advances of this multilayer model and its possible outcomes in biological, physical and robotic sciences.

## 1. Introduction

### 1.1 Modelling collective motion

Collective motion is one of the most ubiquitous collective behaviour displayed by interacting organisms such as cells [1–3], bacteria [4–7], invertebrates (in locusts: [8–10], in ants: [11,12], in honeybees: [13]) and vertebrates (in birds: [14–16], in fish: [17–19], in mammals: [20]) including humans [21,22]. A growing interest to decipher the link between the individual behaviours and the collective patterns has arisen out of these numerous observations and led to the development of models simulating the collective movement of agents inspired from birds, mammals or fish, the latter being the focus of this paper.

Different models exist to describe fish schooling. In the self-propelled particles (SPP) models (synchronous [23,24] or asynchronous [25,26]) firstly used for computer animation [27], the interactions between the fish are mostly composed of a collision avoidance component, an alignment component and a cohesion component [28,29]. Similarly, in the social forces (SF) models, the fish are considered as Newtonian particles subjected to social and physical forces that, respectively, ensure the cohesion of the group and that reflect the interaction (e.g. drag) with the environment [30,31]. The SPP and SF models have inspired several studies in statistical physics that aim to characterize the features of a large number of interacting agents at the collective level [32–36]. Finally, the kinematic models describe the evolution of the trajectories of the fish by a stochastic differential equation [37–40]. This modelling approach has successfully described the movement of fish in a closed environment and is a continuous time formulation of a particular case of random walks (RWs). The RWs describe stochastic trajectories built by successive random steps that can be drawn from a uniform distribution (unbiased RW) or a non-uniform distribution (biased RW). These probabilistic models have a wide range of application, from simulating the Brownian motion of particles to the exploratory patterns of many species including humans [41,42].

### 1.2 Information perception

In all mentioned models of schooling, the agents move according to their conspecifics (position, speed, orientation or a combination). Multiple hypotheses have been proposed to calculate the subset of individuals that influence the motion of a focal fish: the metric perception includes all individuals situated within a defined distance; the topological perception includes the *n*th proximal neighbours; the perception based on Voronoi tessellation includes the fish connected to the focal fish by the Delaunay triangulation. However, while these hypotheses produce a coherent movement of the simulated agents, they are not sufficiently constrained by known biology. Therefore, recent works are based on visual perception [43–46]. In these models, the focal fish does not interact with its neighbours according to their Cartesian coordinates but according to their representation in its visual field.

**Figure 1.**
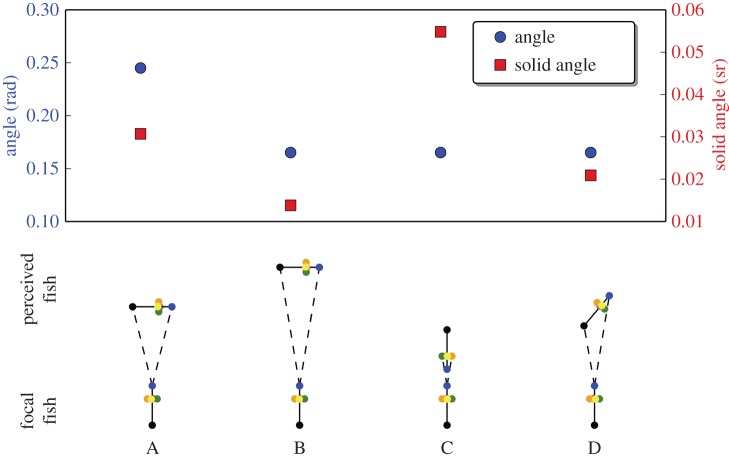
Comparison of the planar angle (blue) and the solid angle (red) captured by a perceived fish in the visual field of the focal fish. In this example, the fish are characterized by a width (1 cm), length (3.5 cm) and height (1 cm). While the planar and solid angles vary according to the distance of the stimuli (A versus B), the solid angle enables the discrimination between situations that are identical according to the planar angle (B versus C versus D).

These theoretical and experimental studies highlighted that the visual sensory system of the fish is determinant for information transfer during collective motions. Indeed, the comparison of the interaction mechanisms showed that a vision-based model outperforms other mechanisms (metric, topologic, Voronoi) in explaining experimental data [45]. In parallel, the increasing knowledge on the visual system of the fish enables the development of models based on sensory systems more coherent with the biological reality. The characterization of the vision of cyprinids reveals that they have a wide visual coverage in the horizontal and the vertical planes and an acute vision in the fronto-dorsal region [47]. To make a step towards more realistic sensory systems, we introduce in this paper a new mechanism based on a perception of three-dimensional stimuli in the visual field. We simulate fish-agents that perceive stimuli (congeners, environment) according to the solid angles—an analogy of the planar angle but in three dimensions—that they intercept in their visual field. The measure of the solid angles enables a more precise discrimination of the stimuli in the perception field than the planar angles. Indeed, by taking into account all dimensions of potential stimuli, the solid angle enables the discrimination between stimuli that are equally perceived by the measure of the planar angle (figure 1).

### 1.3 Information processing

**Figure 2.**
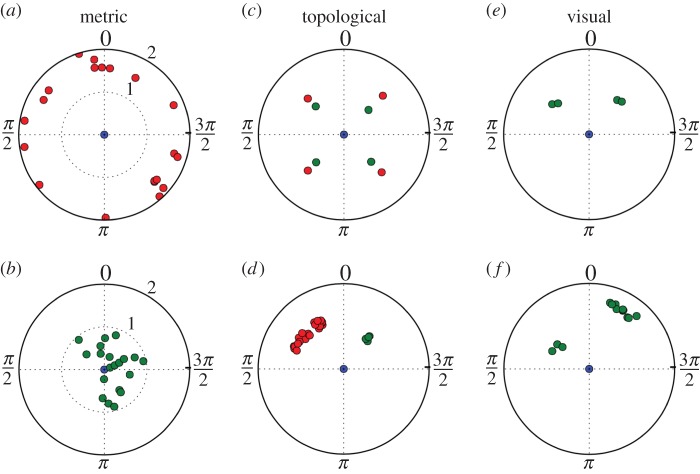
Potential biologically problematic situations in the different models assuming a summation of interaction vectors between the blue focal fish (FF) at the centre and green perceived fish (non-perceived fish are represented in red). In the metric model, the FF perceives no (*a*) or a high number (*b*) of neighbours. In the topological model, the position of the *n*th closest neighbours can be conflicting (*c*) or disproportionate regarding the rest of the group (*d*). Even in visual model based on planar angles, the position of the perceived fish can lead to a resulting vector oriented between the stimuli (*e*) or the number of fish can be overestimated by their captured angle due to proximity (*f*).

Once the focal fish has perceived potential stimuli, this information has to be processed and translated into a movement of the individual. In the SPP and SF models considering only the influence of congeners, a vector of interaction is computed with all neighbours situated in a delimited range (metric models) or according to their proximity rank (topological models). Then, the focal fish moves along the resulting force computed by a weighted summation process of the different interaction forces applied on the focal fish. While it results in a coordinated motion of the agents, these models can also produce biological incoherent behaviours of the simulated individuals: the influence of some congeners can be omitted/overestimated or the resulting vector can point towards a direction where no stimulus is present (figure 2). In addition, these processes have difficulties in reproducing experimentally observed choices between two concurrent stimuli. For example, zebrafish larvae randomly choose to orient towards one of two equidistant sources of light and do not follow the bisector [48]. Here, we present an algorithm to account for information processing by the individuals. Rather than summing influential vectors, we propose to sum probability distribution functions (PDFs) to orient towards the different stimuli.

### 1.4 A multilevel approach

It would be interesting to develop a multilevel modelling approach that takes into account models for the perception and the information processing by the individuals. Beyond the existing SPP and RW models, it requires one to extend the description of the agents by biologically relevant properties. In this paper, we propose a new model at the crossroads of RW and SPP models that includes a probabilistic component (inspired by RW) in the behaviour of the agents that react to their perception field (inspired by SPP models). In the model, the agents choose a direction to move according to a PDF that is determined by the presence of stimuli in their visual perception field. This PDF is computed as a mixture distribution of von Mises distributions centred on each perceived stimulus. Once the PDF has been computed, the direction of the agent is chosen accordingly to this PDF. With this model, we simulate the motion of single individuals or groups of fish evolving in a bounded environment that can include other stimuli like spots of interest.

To validate this model, we measured the individual (single fish) and the collective (group of 10 fish) motion of adult zebrafish in different environmental conditions. We observed the behaviour of zebrafish in a bounded tank with potential spots of interest to take into account the interactions with the environment. First, we analysed the locomotion of isolated individuals swimming in a uniform environment to determine the intrinsic characteristics of their motion (speed, change in orientation and probability of presence). We performed similar measurements in a heterogeneous environment by placing two floating plastic discs acting as attractive spots. Then, we investigated the influence of conspecifics by observing the collective motion of groups of zebrafish in homogeneous and heterogeneous environments. We measured their probability of presence in each location in the experimental tank and their interindividual distances. These experimental data are used for setting the parameters’ values of the individual behaviours as well as the weights of the different types of stimulus on the directional choice of the fish.

## 2. A stochastic model

Our aim is to model fish swimming in nearly two dimensions in a bounded environment with potential spots of interest that attract them. We simulate agents that update their position vector *X*_*i*_ with a velocity vector *V*_*i*_ though a discrete time process in a bounded two-dimensional space:

2.1 $$X_{i}(t+\delta t)=X_{i}(t)+V_{i}(t)\delta t$$

and

**Figure 3.**
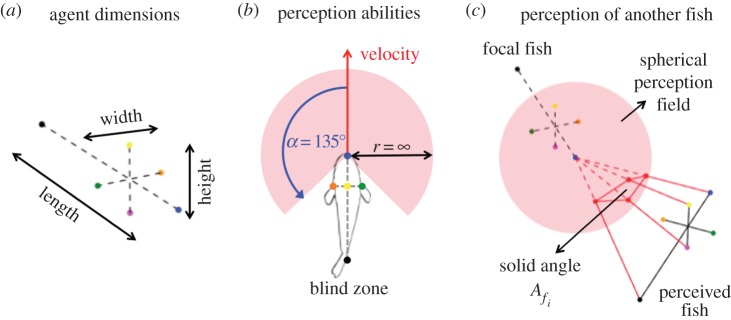
Scheme of the simulated fish and their perception abilities. (*a*) The simulated fish swim on a two-dimensional plane space and are characterized by a width (1 cm), length (3.5 cm) and height (1 cm). (*b*) Their spherical perception field is defined by an angular perception zone and an infinite distance of perception. The position (*x*, *y*) of the fish is updated at each time step by a velocity vector computed according to the perceived stimuli. (*c*) The fish present in their perception field are perceived as the solid angle *A*_*f*_*i*__ captured by their extremities. Similarly, we computed the solid angle *A*_*s*_*i*__ captured by the spots of interest.

2.2 $$V_{i}(t+\delta t)=v_{i}(t+\delta t)\Theta_{i}(t+\delta t)$$

with *v*_*i*_ the linear speed of the *i*th agent and *Θ*_*i*_ its orientation. As we focus on the decision-making process of the fish to choose its orientation in a complex environment (walls of the tanks, spots of interest) with other fish, we assume the simple hypothesis that the linear speed *v*_*i*_ of the agent is randomly drawn from the instantaneous speed distribution measured in our experiments. The novelty of this model is the computation of the orientation *Θ*_*i*_. We model the spherical visual perception field of the fish (figure 3) and describe their probability to move in all potential directions by a circular PDF extending from −*π* to *π*. Thus, *Θ*_*i*_ is not computed as a resulting vector with potential noise but is drawn from a circular PDF formed by von Mises distributions, an equivalent to the Gaussian distribution in circular probability. This PDF is characterized by a measure of location *μ* (equivalent to the mean of a Gaussian PDF) and a measure of concentration *κ* (an inversely proportional equivalent to the variance of a Gaussian PDF). For a fish that perceives no stimulus, the distribution of probability is described by *μ*=0 while the value of *κ*_0_ is determined experimentally (equation (2.3)). By doing so, a fish that perceives no stimulus will move forwards with a deviation inversely proportional to *κ*_0_. As our goal is to model fish moving in a bounded tank, we introduce the interaction of the fish with the walls in the computation of this PDF. As soon as a fish is situated at a distance shorter than a threshold value *d*_w_, we assume that the fish starts a wall-following behaviour. To simulate this behaviour, the value of *μ* is not equal to 0 but to the direction along the followed wall. As there are two potential directions, the PDF is computed as the weighted sum of the PDFs associated with each direction. Thus, the PDF *f*_0_(*θ*) for a fish to move in each potential direction *θ* in a bounded tank without perceptible stimulus is given by

2.3 $$f_{0}(\theta )=\left\{ \begin{array}{ll} \frac{1}{2\pi I_{0}(\kappa_{0})}\exp[\kappa_{0}\cos(\theta )], & \text{if }d\geq d_{w}\\ \frac{1}{2}\sum_{i=1}^{2}\frac{1}{2\pi I_{0}(\kappa_{w})}\exp[\kappa_{w}\cos(\theta -\mu_{w_{i}})], & \text{if }d<d_{w} \end{array}\right.$$

and

2.4 $$I_{0}(\kappa )=\sum_{k=0}^{\infty }\frac{{\left(\kappa /2\right)}^{2k}}{k!\Gamma (k+1)}$$

with *d* the distance to the closest wall, *d*_w_ the threshold distance determining the interaction with the walls, *κ*_0_ and *κ*_w_ the dispersion parameters, respectively, associated with the basic-swimming and wall-following behaviours, *μ*_*w*_1__ and *μ*_*w*_2__ the two possible directions along a considered wall and *I*_0_ the modified Bessel function of first kind of order zero. The values of *d*_w_, *κ*_0_ and *κ*_w_ are determined experimentally.

Equations (2.1)–(2.4) are sufficient rules to simulate a fish swimming in a bounded tank. However, as we aim at simulating groups of fish moving in a homogeneous but also heterogeneous environment, we implemented the interactions with other congeners and spots of interest. As soon as a fish perceives stimuli in its perception field, its PDF is influenced by those stimuli following two steps: information gathering and information processing.

### 2.1 Information gathering

We simulate fish-agents that can perceive and react to three-dimensional stimuli perceived in their visual field. The fish are modelled as three-dimensional polygons with six vertices swimming on a two-dimensional plane space (figure 3*a*). Their visual perception is simulated as a cyclopean vision sensor that has a 270° spherical field of view extending frontally and laterally and an infinite effective radius (figure 3*b*). The fish perceive potential stimuli by the solid angles that the projection of their extremities captures in their spherical perception field (figure 3*c*).

To reflect our experimental conditions, we include two potential stimuli in our simulations: fish and spots of interest. The fish are considered as polygons of length=0.035 m, width=0.01 m and height=0.01 m whose extremities form the boundaries of the fish. The spots of interest are considered as discs with a radius of 0.1 m floating 0.05 m above the plane space.

### 2.2 Information processing

Once all potential stimuli have been perceived, the model computes a PDF for the focal fish to move according to each stimulus (fish or spots). For example, the probability of the focal fish to orient towards a perceived fish is given by a von Mises distribution clustered around this fish:

2.5 $$f_{f_{i}}(\theta )=\frac{1}{2\pi I_{0}(\kappa_{f})}\exp[\kappa_{f}\cos(\theta -\mu_{f_{i}})]$$

and

2.6 $$I_{0}(\kappa_{f})=\sum_{k=0}^{\infty }\frac{{\left(\kappa_{f}/2\right)}^{2k}}{k!\Gamma (k+1)}$$

with *θ* the potential direction of movement of the fish, *μ*_*f*_*i*__ the location of the perceived fish *i* and *κ*_f_ a measure of concentration.

The model computes a distribution for each stimulus (fish or spot) in the perception field of the focal fish. Then for each type of stimuli, it performs a weighted sum of all distributions proportional to the ratio of the solid angle A_*_i__ captured by each stimulus to the sum of the solid angles captured by all stimuli *A*_T_*__. For example, the PDF computed for all perceived fish is given by

2.7 $$f_{F}(\theta )=\sum_{i=1}^{n_{f}}\frac{A_{f_{i}}}{A_{T_{f}}}\frac{1}{2\pi I_{0}(\kappa_{f})}\exp[\kappa_{f}\cos(\theta -\mu_{f_{i}})]$$

and

2.8 $$A_{T_{f}}=\sum_{i=1}^{n_{f}}A_{f_{i}}$$

with *A*_*T*_f__ the sum of the solid angles *A*_*f*_*i*__ captured by each fish *i* and *n*_f_ the number of perceived fish.

We calculate a similar PDF for the spots of interest perceived by the focal fish:

2.9 $$f_{S}(\theta )=\sum_{i=1}^{n_{s}}\frac{A_{s_{i}}}{A_{T_{s}}}\frac{1}{2\pi I_{0}(\kappa_{s})}\exp[\kappa_{s}\cos(\theta -\mu_{s_{i}})]$$

and

2.10 $$A_{T_{s}}=\sum_{i=1}^{n_{s}}A_{s_{i}}$$

with *μ*_*s*_*i*__ the location of the centre of the perceived spot *i*, *κ*_s_ the dispersion parameters associated with the spots, *A*_*T*_s__ the sum of the solid angles *A*_*s*_*i*__ captured by each spot and *n*_s_ the number of perceived spots.

**Figure 4.**
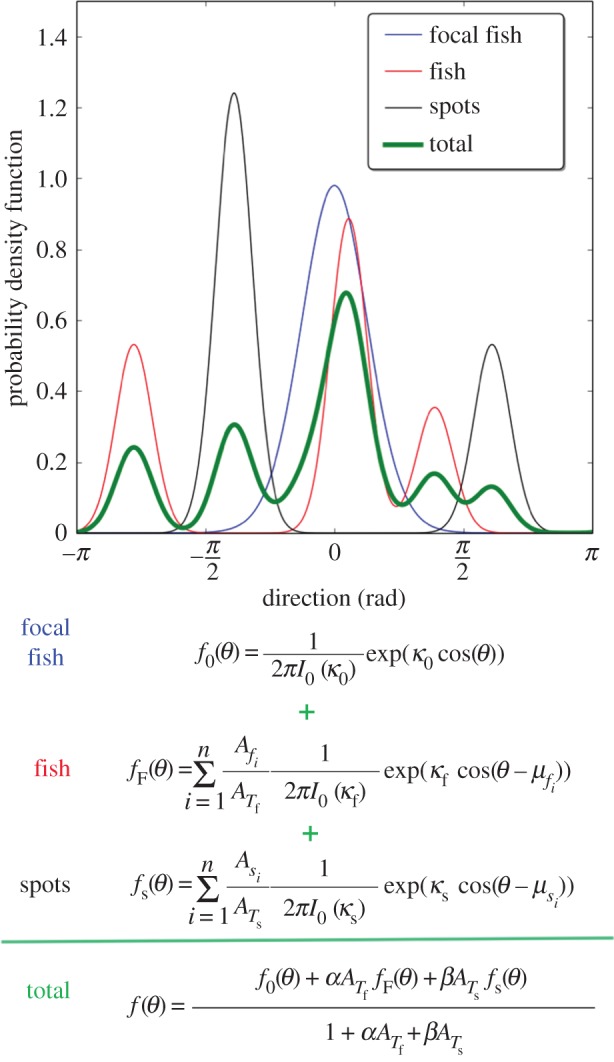
Weighted sum of the three PDFs calculated for the focal fish according to the congeners and the shelters perceived by the individualto compute its final PDF. The direction taken by a fish is drawn randomly from the final computed PDF (in green) by inverse transform sampling.

Once the PDFs have been computed for each type of stimuli (fish and/or spots), we calculate a weighted sum of the PDFs to obtain the global probability distribution function *f*(*θ*) of the focal fish to move towards a given direction. In this first approach, we assumed that the weight of the PDF associated with each type of stimuli is a linear function of the total solid angle *A*_T_*__ that they capture in the perception field of the focal fish (figure 4). This implies that the fish respond more strongly to largely perceived stimuli but it also allows a potential hierarchy in the response to different stimuli. Thus, the global PDF *f*(*θ*) is given by

2.11 $$\begin{array}{rl} f(\theta ) & =\frac{f_{0}(\theta )+\alpha_{*}A_{T_{\;\;f}}f_{F}(\theta )+\beta_{*}A_{T_{s}}f_{S}(\theta )}{1+\alpha_{*}A_{T_{\;\;f}}+\beta_{*}A_{T_{s}}} \end{array}$$

2.12 $$\begin{array}{rl} \alpha_{*} & =\left\{ \begin{array}{ll} \alpha_{0}, & \text{if }d\geq d_{w}\\ \alpha_{W}, & \text{if }d<d_{w} \end{array}\right. \end{array}$$

2.13 $$\begin{array}{rl} \;\text{and}\;\beta_{*} & =\left\{ \begin{array}{ll} \beta_{0}, & \text{if }d\geq d_{w}\\ \beta_{W}, & \text{if }d<d_{w} \end{array}\right. \end{array}$$

with *α*_0_ and *β*_0_ the parameters weighting the influence of, respectively, the fish and the shelters for a fish distant from any wall and *α*_W_ and *β*_W_ for a fish following a wall. These parameters are fitted experimentally.

Then, we numerically compute the cumulative distribution function corresponding to this custom PDF *f*(*θ*) by performing a cumulative trapezoidal numerical integration of the PDF in the interval [−*π*,*π*]. Finally, the model draws a random direction *Θ*_*i*_ in this distribution by inverse transform sampling. The position of the fish is then updated according to this direction and this velocity with equations (2.1) and (2.2). All parameters of the model are listed in table 1 with their values fitted from experimental data as well as a short description.

**Table 1.** List of parameters.

parameter	value	description
*d*	—	distance to the closest wall
*d*_w_	0.05 m	distance of interaction with the wall
*μ*_w_*__	—	direction along a wall
*μ*_f_*__	—	location of a perceived fish
*μ*_s_*__	—	location of a perceived spot
*κ*_0_	6.3	dispersion parameter associated with basic-swimming
*κ*_w_	20	dispersion parameter associated with wall-following
*κ*_f_	20	dispersion parameter associated with a fish
*κ*_s_	20	dispersion parameter associated with a spot of interest
*A*_f_*__	—	solid angle captured by a fish
*A*_*T*_f__	—	sum of the solid angles captured by all fish
*A*_s_*__	—	solid angle captured by a spot of interest
*A*_*T*_s__	—	sum of the solid angles captured by all spots of interest
*α*_0_	55^a^	weight of the perceived fish during basic-swimming
*α*_w_	20^a^	weight of the perceived fish during wall-following
*β*_0_	0.15^b^	weight of the perceived spots during basic-swimming
*β*_w_	0.01^b^	weight of the perceived spots during wall-following
*w*_F_	$\frac{1}{2}$	factor of weight for *α*_0_ and *α*_w_ when fish and spots are present
*w*_S_	$\frac{1}{9}$	factor of weight for *β*_0_ and *β*_w_ when fish and spots are present

^a^Value fitted when only other fish are present.

^b^ Value fitted when only spots of interest are present.

## 3. Results

### 3.1 Homogeneous environment

**Figure 5.**
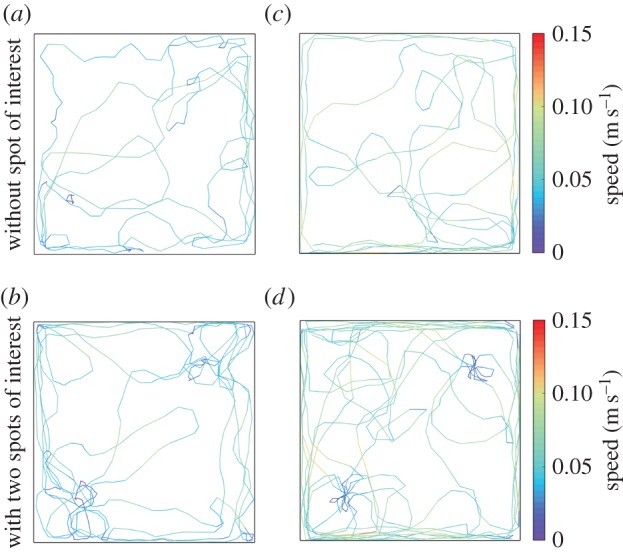
Example of experimental (*a*,*b*) and simulated (*c*,*d*) trajectories of a fish swimming alone during 10 min in the absence (*a*,*c*) orpresence (*b*,*d*) of two floating discs. The colour of the trajectory indicates the speed of the individual.

We measured the positions of ten fish swimming individually in our 1.20×1.20 m experimental tank during 1 h. Based on this tracking, we built the trajectories of the fish and computed their speed and change in orientation. An example of a 10 min trajectory of a fish is given in figure 5*a*. The fish swim at an average speed of 0.07±003 m s^−1^ and are mainly moving forwards with soft changes of orientation (average change=−0.03±0.84 rad, see electronic supplementary material, figure S1). The distribution of the positions detected in the tank shows that the higher probability of the presence is situated along the walls and that the fish avoid the centre of the tank (figure 8*a*).

**Figure 6.**
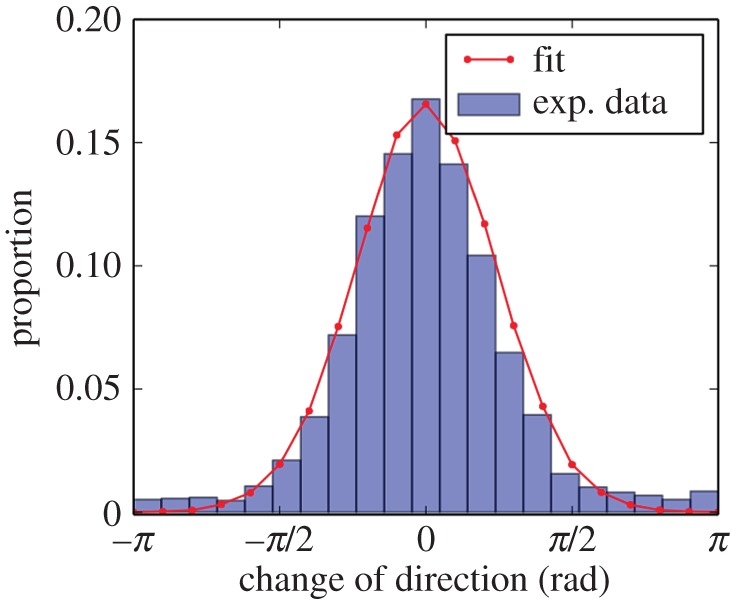
Experimental distribution and fitting by sum of least-squares minimization of direction changes of single zebrafish swimming in a homogeneous tank. Experimental distribution (blue) and fitting (red) of changes ofdirection obtained for *κ*_0_=6.3. The data were calculated for trajectories measured at least at 0.3 m from any walls to minimize their influence on the fish orientation.

We used this experimental data to set the parameters of our model in order to simulate the movement of a single fish in a homogeneous tank. In the simulations, the speed of the agent is drawn from the experimental distribution of the instantaneous speed of the fish. Speeds are drawn independently from each other so that there is no correlation between the speed of agent *i* at time *t* and *t*+1. While this differs obviously from the reality, we do not take into account speed matching in this first parametrization of our model for simplicity. The experiments with single fish allow us to fit the parameter value characterizing the change of direction of fish *κ*_0_. To do so, we measured the change of direction of the fish when they were at least at 0.30 m from any walls. By doing so, we excluded the potential influence of the walls and considered only the intrinsic changes of direction. Then, we compared these experimental distributions to theoretical ones and selected the best fitting. In this first approach, we chose the sum of least-squares minimization as a rapid and low computational cost fitting method. The best fitting of this experimental distribution was given by *κ*_0_=6.3 (electronic supplementary material, figure S2), as shown in figure 6.

For the experiments involving a single fish, only the interaction with the walls of the tank is present. Therefore, the other relevant parameters are the distance of interaction with the walls *d*_w_ and the measure of concentration *κ*_w_ of the PDFs associated with wall following. To estimate these parameter values, we performed simulations with different couples of values (*d*_w_, *κ*_w_) and compared the experimental distributions of change in orientation and of probability of the presence with those generated by the simulations. The fitting of these parameters showed that *d*_w_=0.05 and *κ*_w_=20 are the best values to reproduce our experimental data (electronic supplementary material, figure S3). A 10 min trajectory of a simulated fish with these parameter values is shown in figure 5*c*.

**Figure 7.**
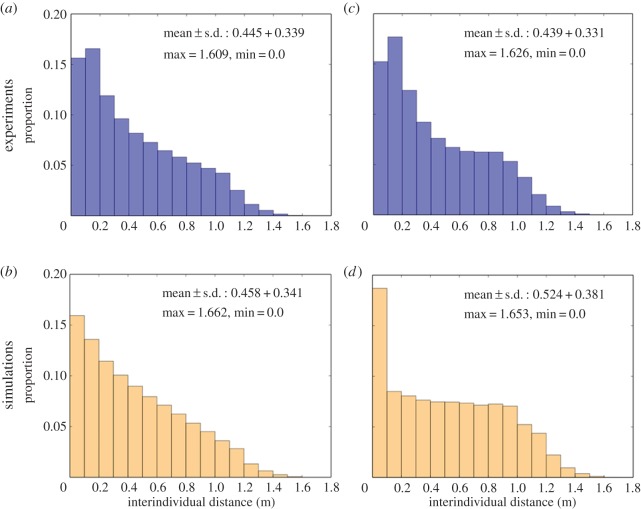
Interindividual distances measured between all pairs of the 10 fish swimming in a homogeneous environment (*a*,*b*) or in theof two spots of interest (*c*,*d*). Results are obtained for 10 experiments of 1 h with 10 groups of 10 fish (*a*,*c*) and 10 simulations with 10 agents (*b*,*d*).

**Figure 8.**
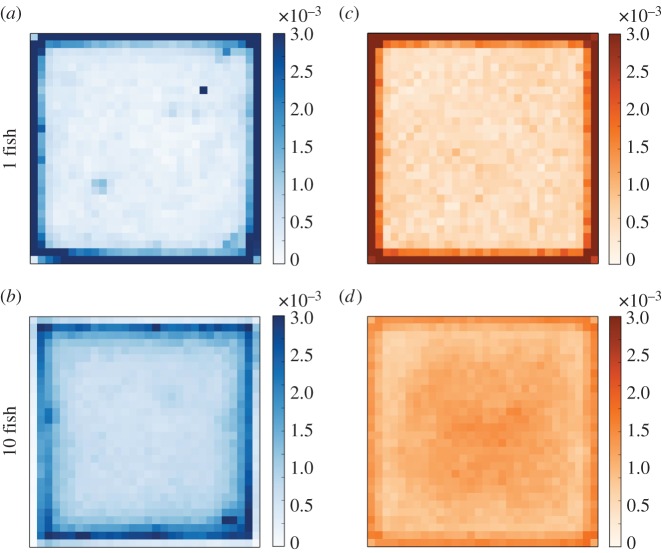
Probability of the presence for experimental (*a*,*b*) and simulated (*c*,*d*) data. Results are obtained for 10 replicates of 1 h. (*a*) Experimental individual behaviour of single fish from AB strain in the empty experimental tank. The probability of the presence showed that fish were mainly swimming along the walls of the tank. (*b*) Experimental results obtained for groups of 10 AB strain zebrafish in a homogeneous environment. Similar to singlefish, groups of 10 zebrafish were mainly swimming along the walls of the tank. (*c*) Probability of the presence of a simulated single fish in a homogeneous environment during 1 h (results for 10 simulations). (*d*) Probability of the presence of a simulated group of 10 fish in a homogeneous environment during 1 h (results for 10 simulations).

Then, we performed experiments with 10 groups of 10 zebrafish. As observed for single fish experiments, fish were mostly detected along the walls of the tank (figure 8*c*). We measured the distance between all pairs of fish at each time step to characterize the cohesion of the group. The distribution of these interindividual distances shows an average of 0.394 m±0.38 m with the mode of the distribution between 0.1 and 0.2 m (figure 7*a*).

To simulate experiments with 10 fish, we introduce three parameters describing the interaction with the fish: the measure of concentration *κ*_F_ associated with the PDF computed for each perceived fish and the parameters *α*_0_ and *α*_W_ that weight the influence of other individuals on a fish that is far from (*α*_0_) or close (*α*_W_) to a wall. To reduce the parameters’ space and the complexity of the fitting, the value of *κ*_F_ is assumed to be similar to *κ*_w_ and equal to 20. By doing so, we consider that a fish orients towards a given target *μ* with a high accuracy. To determine the value of the weights α_*_ we perform simulations with different values of (*α*_0_, *α*_W_) and compare the distributions of the interindividual distances and the probability of the presence with those obtained for the experiments. The best values to reproduce both distributions are *α*_0_=55 and *α*_W_=20 (electronic supplementary material, figure S4). Thus, the fish that follow a wall are less influenced by other congeners than the fish situated in the centre of the tank. With these parameter values, the model reproduces the probability of the presence displayed by groups of 10 zebrafish as shown in figure 8*d*. Concerning the distribution of interindividual distances, the model reproduces the decreasing distribution observed in the experiments except for the mode of the distribution that is between 0 and 0.1 m (figure 7*b*).

### 3.2 Heterogeneous environment

**Figure 9.**
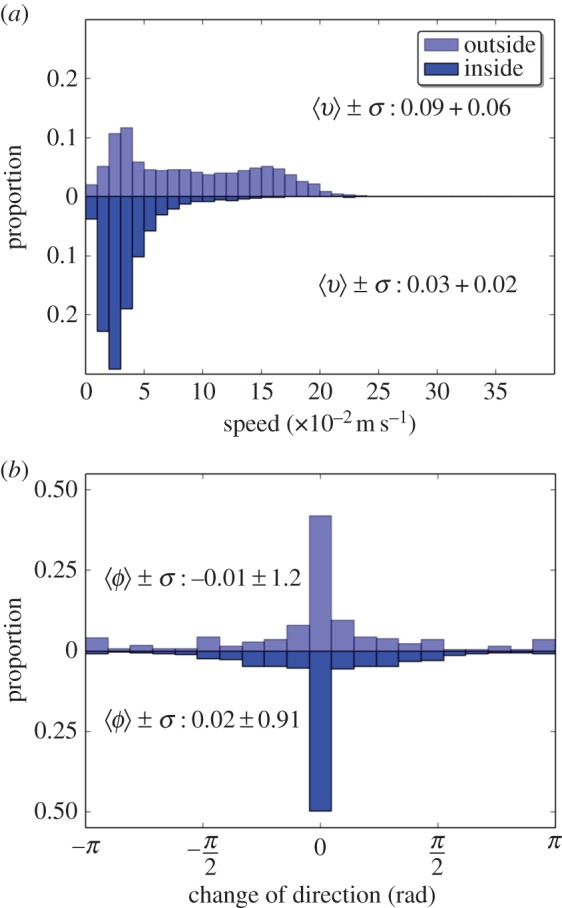
Experimental individual behaviour of single fish from AB strain in the experimental tank with two spots of interest. (*a*) The distribution of the speed shows an average speed of 0.09±0.06 m s^−1^ outside the spots whilefish were swimming with an average speed of 0.03± 0.02 m s^−1^ under the spots. (*b*) The distribution of the change in orientation highlights that fish are mainly swimming forward with low deviation both outside and inside the spots. Results are pooled for 10 replicates of 1 h.

**Figure 10.**
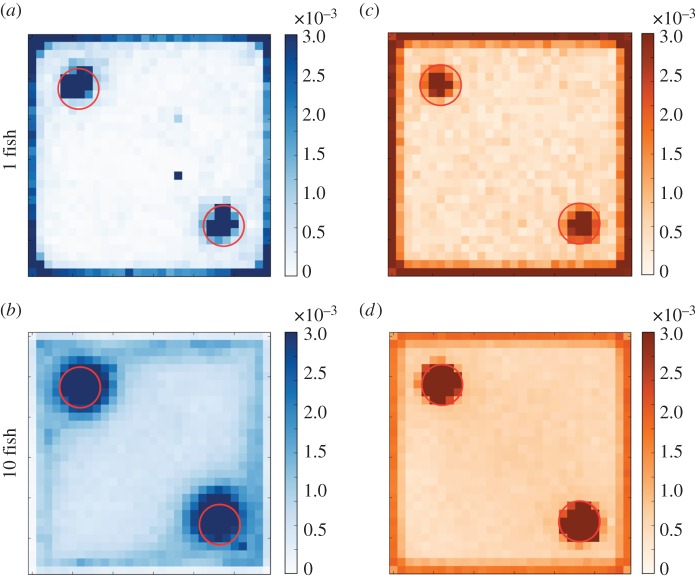
Probability of the presence for experimental (*a*,*b*) and simulated (*c*,*d*) data. Results are obtained for 10 replicates of 1 h. (*a*) Experimental individual behaviour of a single fish from AB strain in a tank with two floating discs. The probability of the presence showed that fish were mainly swimming along the walls or under the discs. (*b*) Experimental results obtained for groups of 10 AB strain zebrafish in a heterogeneous environment.The probability of the presence showed that fish were mainly present under the floating discs. Results are obtained from 10 replicates of 1 h. (*c*) Probability of the presence of a simulated single fish in a homogeneous environment during 1 h (results for 10 simulations). (*d*) Probability of the presence of a simulated group of 10 fish in a homogeneous environment during 1 h (results for 10 simulations).

We added two spots of interest placed at $25\sqrt{2}\;\mathrm{cm}$ from two opposite corners along the diagonal of the tank. These spots consisted of blue plastic discs (20 cm in diameter) floating at the water surface and hung by nylon threads. An example of path followed by a fish during 10 min is shown in figure 5*b*. We calculated similar parameters from the individual positions of the fish (10×1 fish) moving alone in the tank. In the presence of two spots, the fish are mainly detected along the walls and under the spots as shown by their probability of the presence (figure 10*a*). The experiments with single fish also showed that the fish decrease their speed under these floating discs. Indeed, the separation of the speed distribution measured *outside* or *inside* the spots shows that the average speed was three times slower when the fish were located under a floating disc (figure 9*a*). On the contrary, the presence of the spots did not affect the distribution of the changes in orientation that were similar *outside* and *inside* (figure 9*b*).

Only the interaction with the walls and the shelters of the tank are present in the experiments involving a single fish. As *d*_w_=0.05 and *κ*_w_=20 were fitted by our experiments in homogeneous environment, we explore the parameter values of *β*_0_ and *β*_W_ (the weight of influence of the spots) and assume that the measure of concentration associated with the spots *κ*_s_=*κ*_w_=20. We perform simulations for different values of (*β*_0_,*β*_W_) and compare their results with the experimental probability of the presence and distribution of change of orientation. The best values to reproduce our experimental data are *β*_0_=0.15 and *β*_W_=0.01 (see the electronic supplementary material). As observed for the influence of the congeners, these values indicate that the fish following a wall are less influenced by the spots than the fish swimming in the centre of the tank. With these parameter values, the model reproduces the spatial distribution of the fish along the walls and under the floating discs (figure 10*c*).

Finally, we observed 10 groups of 10 zebrafish swimming in the presence of two spots of interest during 1 h. In this case, the fish were also observed mainly under the spots and along the walls but showed a preference for the spots (figure 10*b*). The measure of the interindividual distances shows that the presence of spots does not have a strong influence on the distance between the fish (figure 7*c*). Indeed, the average distance between the individuals is 0.439±0.331 m, which is 6 mm less than observed in the absence of floating discs.

We simulated these experiments with groups of 10 zebrafish with our model by integrating the interactions of the agents with the other fish and the spots of interest. In the previous experiments, the parameters ruling the interactions with the fish (*α*_0_, *α*_W_) and the spots of interest (*β*_0_, *β*_W_) were fitted independently but here, both stimuli are simultaneously present in the perception field of the fish. Therefore, we investigate the relative importance of both stimuli by weighting the influence of the congeners and the spots. To do so, we multiply the previously fitted values of (*α*_0_, *α*_W_) and (*β*_0_, *β*_W_) by different weighting factors $w_{F}=\{\frac{1}{1},\frac{1}{2},\ldots ,\frac{1}{10}\}$ and $w_{S}=\{\frac{1}{1},\frac{1}{2},\ldots ,\frac{1}{10}\}$. For example, values $w_{F}=\frac{1}{1}$ and $w_{S}=\frac{1}{1}$ imply that the two stimuli are simply added, whereas values $w_{F}=\frac{1}{2}$ and $w_{S}=\frac{1}{5}$ imply that the influence of the fish is divided by 2 and the influence of the spots by 5. We perform simulations for each couple of (*w*_F_,*w*_S_) and compare the distributions of the probability of the presence and the interindividual distances with those measured experimentally. The best fit is given by $w_{F}=\frac{1}{2}$ and $w_{S}=\frac{1}{9}$ (electronic supplementary material, figure S6), which implies that the influences of the fish and the spots have to be decreased by a factor 2 and 9, respectively. Therefore, when both stimuli (congeners and spots) are perceived by the fish, the best values of α_*_ and β_*_ to reproduce the experimental data are *α*_0_=27.5, *α*_W_=10, *β*_0_=0.016 and *β*_W_=0.0011. While these values give a correct fit of the experimental probability of the presence (figure 10*d*), the model does not perfectly reproduce the distribution of the interindividual distances (figure 7*d*). Similar to the groups of fish in a homogeneous environment, the mode of the interindividual distances measured in the simulation is between 0 and 0.1 m, whereas the mode of the experimental distances is between 0.1 and 0.2 m.

## 4. Discussion

### 4.1 Experimental results

In this study, we observed the individual and collective behaviour of zebrafish in homogeneous and heterogeneous environments. Firstly, we observed single zebrafish swimming in our experimental tank to characterize their swimming behaviour. In a homogeneous environment, the fish were mainly following the walls of the tank and avoided the centre of it. This observation was also reported in studies performed with zebrafish and robotic-fish [49]. We found an average speed of 0.07±0.03 m s^−1^ and an average change of orientation of −0.03±0.84 rad, in coherence with previous studies performed on single zebrafish individuals [39,40,50]. Our experiments with 10 zebrafish showed that the individual space usage when in a group did not differ substantially from space usage when alone. The fish in groups were also mainly detected along the walls of the tank.

The presence of floating discs influences the spatial distribution of the fish in the experimental tank. The shaded area seemed as attractive as the walls because the fish showed a similar probability of the presence near both stimuli. These discs had no influence on the change of direction of the fish but resulted in a spatial differentiation of the instantaneous speed of the individuals. Indeed, the fish swam with an average speed of 0.09±0.06 m s^−1^ outside the shelters but with an average speed of 0.03±0.02 m s^−1^ under them. Such reduction of speed can indicate that shaded areas are potentially considered as temporary resting sites by the fish. The observation of groups of 10 fish showed similar results with preference for both the walls and the shaded areas.

These results highlight that the zebrafish are avoiding free water and prefer to swim near potential shelters (floating objects or bank). Although the fish were attracted by the floating disc, they did not seem to avoid the light because their presence under the disk and along the wall (that are exposed to light) are similar. These observations are in accordance with the ecology of the species that shows a diurnal activity in Nepalese and Indian shallow water and rice paddies [51].

### 4.2 A hybrid modelling approach

Based on our observations and on the literature, we developed a model describing the individual behaviour of fish during collective motion. This model is based on a three-dimensional perception system accounting for the vision of zebrafish in which the objects are described by the solid angles that they capture in their perception field. By doing so, we are closer to a realistic description of the sensory system of the fish. In this first step, we assumed the simple hypothesis that the objects were homogeneously perceived in the three-dimensional sensory field. However, the recent characterization of the visual system of zebrafish highlighted the existence of centres of acute vision (i.e. *areae*) in the fronto-dorsal region [47]. Therefore, the future models should take into account this heterogeneity in the perception field. In addition, the position of the areae differs from one species to another and is expected to influence the structure of the fish school [47]. The extension of our model to three-dimensional movements would allow one to test this hypothesis.

**Figure 11.**
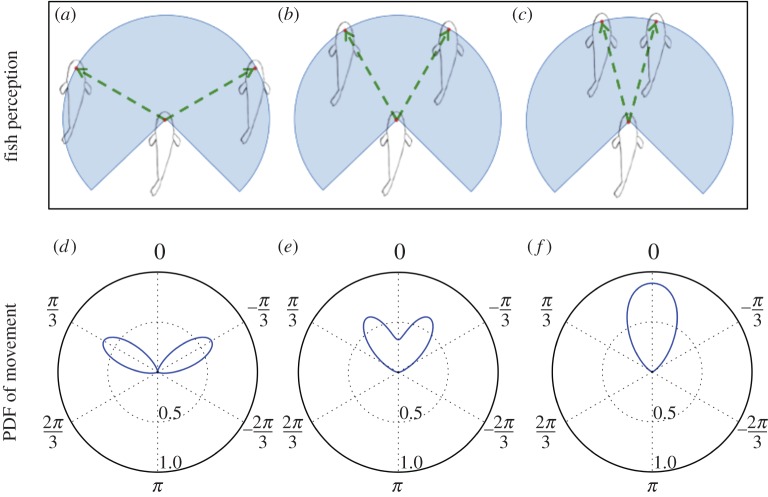
Evolution of the normalized PDF *f*_F_(*θ*) describing the influence of the neighbouring fish according to their position. (*a*,*d*) Two neighbours far from each other will generate two distinct peaks in the PDF, leading to a choice for the focal fish to orient preferentially towards one of them. (*b*,*e*) If the neighbours get closer to each other, the two peaks start to merge and thefocal fish has a non-zero probability to move forwards. (*c*,*f*) Finally, if the neighbours are close to each other, the two peaks completely merge into a unique one pointing between both neighbours. In this latter case, the focal fish has a higher probability to orient between the two perceived neighbours. Thus, by summing the PDFs rather than interaction vectors, our algorithm for individual decision-making spontaneously produces transitions from choice to compromise (and vice versa) according to the spatial distribution of the perceived stimuli.

In addition to this sensory system, we proposed a stochastic mechanism to determine the direction of an agent according to its visual field. Rather than computing a resulting vectorial force that is applied to the fish, we described the decision-making process of the fish to make an intentional movement according to its perception field. In this first step, we represented the choice of the individual by a stochastic process. The fish can potentially move in any direction, but it will favour directions associated with a perceived stimuli (e.g. congeners). This is made possible by the representation of the perception field of the fish and its translation into the normalized sum of the PDFs associated to each perceived stimulus. With this approach of PDFs’ summation, we can potentially include all kinds of stimuli (congeners, environment, food, etc.) that are perceived by the fish and account for a choice or a compromise between potentially antagonist stimuli. Indeed, two stimuli distant from each other will generate two distinct peaks in the PDF, leading to a choice of the fish to orient preferentially towards one of the stimuli (figure 11*a*,*d*). Such choice between two concurrent stimuli has been evidenced in zebrafish larvae that orient towards one of two light sources rather than swimming towards their bisector [48]. On the contrary, when the stimuli get closer to each other (figure 11*b*,*e*), the two peaks of probability merge into a unique one pointing between both stimuli (figure 11*c*,*f*). In this case, the fish has a higher probability to orient between the stimuli rather than choosing one of them. Thus, the proposed algorithm is able to spontaneously transit from choice to compromise and vice versa according to the spatial distribution of the stimuli.

Moreover, most of the models consider motion in an unbounded homogeneous space (i.e. in a torus geometry). While this assumption is reasonable to study the collective behaviour of animals living in pelagic waters, the interactions with environmental features cannot be neglected for the species living in small streams. Therefore, our model takes into account a bounded and heterogeneous space to be closer to the natural conditions of *Danio rerio*. The model correctly reproduces the behaviour of single individuals swimming in a bounded aquarium. The fish mainly follow the walls but sometimes swim in the centre of the tank. The probability of the presence of the fish is also correctly reproduced when simulating groups of 10 agents. However, the distribution of interindividual distances is biased towards short distances. This could originate from the absence of a preferred distance or an avoidance distance between the agents. Indeed, while the agents of the model can overlap, the fish have to respect a distance between them. Such distance was not introduced in this first parametrization of the model to limit the number of parameters. In a heterogeneous environment, the model was also able to reproduce the individual trajectories of single fish that transit between the walls and the spots of interest. As in the homogeneous environment, the probability of the presence of the groups of fish is also reflected in the simulations, but again, the distribution of the interindividual distances is biased towards short distances. These results indicate that further versions of the model should include additional biological variables that can be species dependent like a preferred distance.

In this study, we applied our decision-making algorithm to an individual-based model (IBM). Recently, a kinetic model (KM) was proposed to account for the individual movement of isolated zebrafish [39,40]. This model inspired by Gautrais *et al.* [37,38] took into account a dynamic speed regulation characterizing the *D. rerio* locomotion. In our model, we did not implement a function accounting for speed modulation in order to focus on the directional decision-making mechanism. The instantaneous speed was drawn from the experimental distribution with a time step corresponding to the tail-beat period of zebrafish. Then, future work should investigate the impact of the proposed stochastic mechanism for orientation on such KMs. Moreover, the development of a KM version of our IBM could be an intermediate step towards a continuum model description of the group of agents. This could then be used to perform large-scale analysis and predictions of the collective behaviour displayed by very large populations. Such multiscale modelling approach would allow us to identify the properties of the group that are preserved at all scales of analysis or on the contrary that are specific to a particular level of observation [52].

In parallel to the understanding of information processing by the animals, this approach is also a new step towards bioinspired algorithms that can be implemented in robotic agents. Indeed, it is a major scientific challenge to build artificial systems composed of robots that can perceive, communicate to, interact with other agents (biological or robotic) and adapt to their environment [53]. To do so, we need to develop artificial agents that communicate through appropriate channels corresponding to specific animal traits but also that correctly perceive and interpret the signals emitted by the animals [54–56]. In fish, an increasing number of studies aim at developing robotic agents to interact with group of fish [57–60]. Current experiments generally involve one robot that follows a predetermined trajectory or that moves according to the position of fish detected through the intermediary of a camera that films the entire set-up. The development of fully autonomous integrated lures in fish schools (or other species) requires the development of perception abilities for the robotic agents as well as adapted behavioural algorithms. In this perspective, the development of perception-based models is a necessary step towards intelligent artificial systems capable of closing the loop of interaction between the animals and the robots.

## 5. Material and methods

### 5.1 Animals and housing

We acquired 150 adult wild-type zebrafish (*D. rerio* AB strain from Institut Curie, Paris). Fish were 18 months old at the time of the experiments. We kept fish under laboratory conditions, 27°C, 500 μS salinity with a 10 L:14 D cycle. The fish were reared in 55 l tanks and were fed two times per day (Special Diets Services SDS-400 Scientific Fish Food). Water pH was maintained at 7.5 and nitrites (NO^2−^) were below 0.3 mg l^−1^.

### 5.2 Experimental set-up

We recorded the behaviour of zebrafish in a 120×120×30 cm experimental tank made of glass with internal walls covered with white adhesive. The water depth was kept at 10 cm during the experiments. A Logitech^r^ HD Pro Webcam C920 was mounted 160 cm above the water surface to record experiments at a resolution of 1920×1080 and at 15 FPS. The camera was connected to a workstation Dell^r^ Precision T5600 dedicated to the recording of the videos and their analysis. One halogen lamp (450 W) was placed at each corner of the tank and oriented towards the wall to provide indirect lightning of the tank. The whole set-up was confined behind white sheets to isolate experiments and homogenize luminosity.

### 5.3 Experimental procedure

We recorded the behaviour of zebrafish swimming in our tank during 1 h. We tested two numbers of individuals (single fish or groups of 10 fish) in two environmental conditions (homogeneous or heterogeneous). The heterogeneous environment was created by adding two floating discs made of blue coloured Plexiglas (200 mm diameter and 3 mm thick) suspended by nylon threads. The two spots were spaced from 70 cm and located on a diagonal of the square. Before the trials, fish were placed with a hand net in a cylindrical arena (20 cm diameter) made of Plexiglas placed in the centre of our experimental tank. Following a 5 min acclimatization period, this arena was removed and fish were able to freely swim in the experimental tank. We performed 10 replicates for each combination of parameters (number of fish×environmental condition).

### 5.4 Data analysis

The recorded videos were analysed offline using a custom Matlab script developed to detect the position of the fish. This script performed a background subtraction on each frame and transformed it in a binary image according to a pixel intensity threshold given by the user. The software then identified the blobs on this image and kept only the blobs that were formed by more than 20 and less than 200 pixels (those values were obtained by manually identifying the fish on multiple frames). As this method did not allow a perfect detection of all the individuals, we developed a second script that was run after the first one and that plotted the frame where a fish (or more) was undetected for the user to manually identify the missing individual(s). While this analysis tool is time-costly, it allowed us to identify the fish that were partially hidden during a collision/superposition with another fish or the fish that were situated under the floating discs as our program was not able to detect them due to a lack of intensity of the pixel after the background subtraction. The positions *P*(*x*,*y*) of the fish were recorded at each time step $t=\frac{1}{15}\;s$ during the experiment with single fish in homogeneous environment and *t*=1 s for all other experimental conditions. This allowed us to build the trajectory of each individual for experiments involving a single fish and to compute the speed of the individuals as well as their change in orientation between successive positions. The instantaneous speed *v*_*t*_ was calculated on three positions and thus computed as the distance between *P*(*x*,*y*,*t*−1) and *P*(*x*,*y*,*t*+1) divided by two time steps, while change in orientation was computed as the angle between two successive speed vectors. The distributions of speed were computed only for parts of the trajectory during which the fish were not in freezing behaviour (i.e. immobile). This corresponds to a spontaneous speed higher than 1 mm s^−1^. As our tracking system could switch the identity of the fish after occlusions (i.e. superposition of two indistinguishable individuals), we did not calculate individual measures for data obtained with groups of fish but characterized the aggregation level of the group.

### 5.5 Implementation and numerical simulations

The model was implemented in a Matlab script. The simulations were run during 10 800 time steps, each time step representing an increment of $\frac{1}{3}$ s to simulate a total time of 1 h, similar to our experiments. This time step was chosen according to the tail beat frequency of the zebrafish of ∼2.5 Hz. By doing so, we assume that zebrafish can potentially change their orientation at each tail beat. The position of the agents on the two-dimensional plane space is described by the position of their head (*x*, *y*, 0). The positions of the other vertices are computed according to the position of the head and the direction of the fish. To compute the solid angle of the perceived stimuli in the perception field of the focal fish, we calculated the area of the spherical polygon delimited by the projection of the stimuli’s vertices on a unit sphere centred on the focal fish. To do so, we divided the polygon in two spherical triangles and computed their spherical excess using L’Huilier’s theorem:

5.1 $$\tan(\frac{1}{4}E)=\sqrt{\tan(\frac{1}{2}s)\tan[\frac{1}{2}(s-a)]\tan[\frac{1}{2}(s-b)]\tan[\frac{1}{2}(s-c)]}$$

with *a*, *b* and *c* the length of the arcs between the vertices expressed in spherical coordinates (*ρ*, *θ*, *ϕ*) and computed by

5.2 $$a=\arccos(\sin(\phi_{1})\sin(\phi_{2})+\cos(\theta_{1}-\theta_{2})\cos(\theta_{1})\cos(\theta_{2})$$

and *s* the semi-parameter given by

5.3 $$s=\frac{a+b+c}{2}.$$

## Supplementary Material

S1 Supplementary figures

## Data accessibility

All experimental data are available in a permanent repository on Dryad (http://dx.doi.org/10.5061/dryad.p2g11) [61].
