# Supplementary material for: A stochastic vision-based model inspired by zebrafish collective behaviour in heterogeneous environments
Source: R Soc Open Sci. 2016 Jan 13;3(1):150473. doi: 10.1098/rsos.150473 (PMC4736928; doi:10.1098/rsos.150473)
Supplement: S1 Supplementary figures [file rsos150473supp1.pdf]

Supplementary material

Supplementary figures of "A stochastic vision based model inspired by zebrafish collective behaviour in heterogeneous environments"

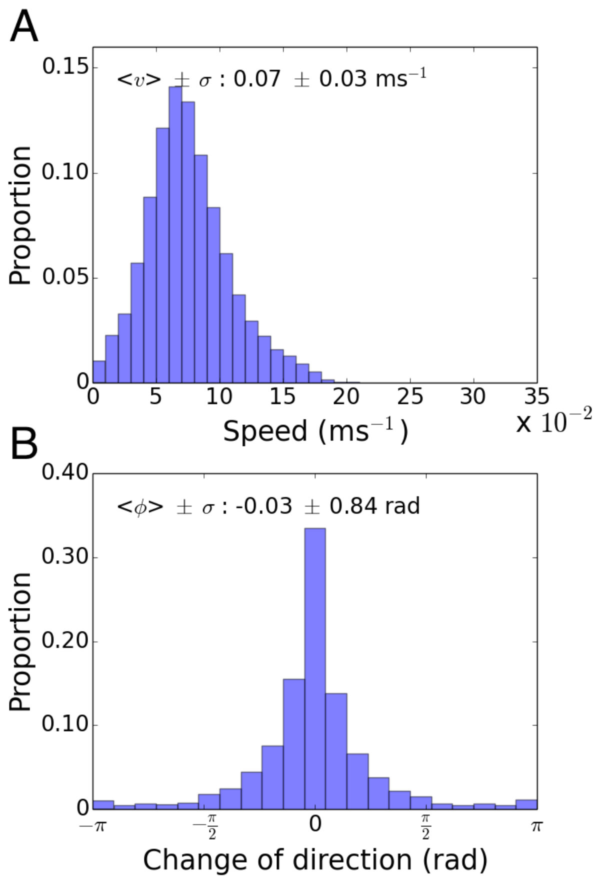

Figure 1: Experimental individual behaviour of single fish from AB strain in the experimental tank without stimuli. (A) The distribution of the speed shows an average speed of 0.07 ± 0.03 ms^{-1}. (B) The distribution of the change in orientation highlights that fish are mainly swimming forward with low deviation. Results are cumulated for 10 replicates of one hour.

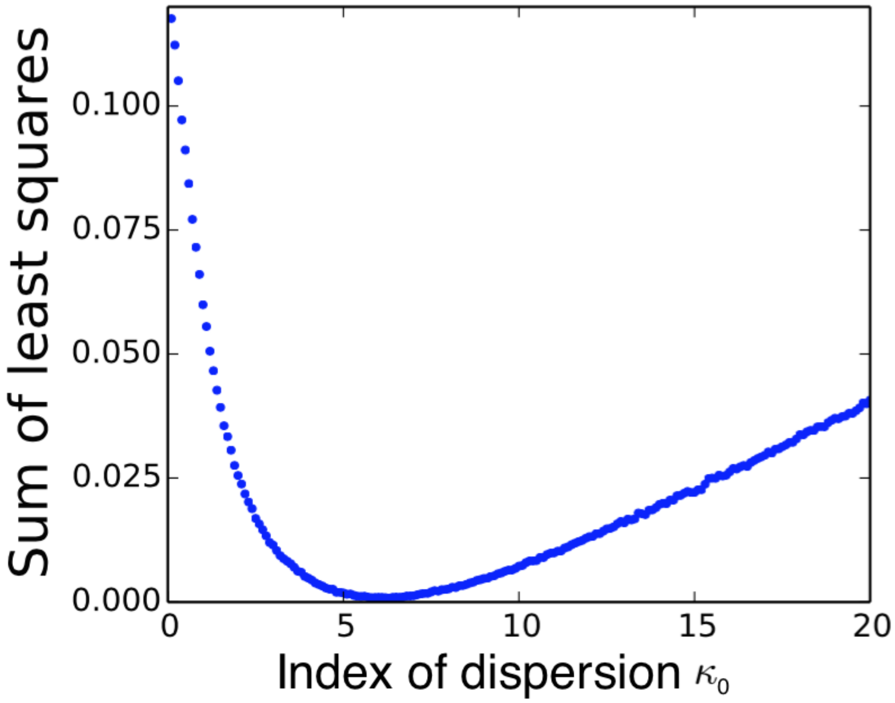

Figure 2: Experimental distribution and fitting by sum of least squares minimisation of direction changes of single zebrafish swimming in a homogeneous tank. Evolution of the sum of the mean squares between the experimental distribution and a theoretical distribution of 100,000 random draws in a von Mises distribution with parameters $\mu = 0$, κ_0.

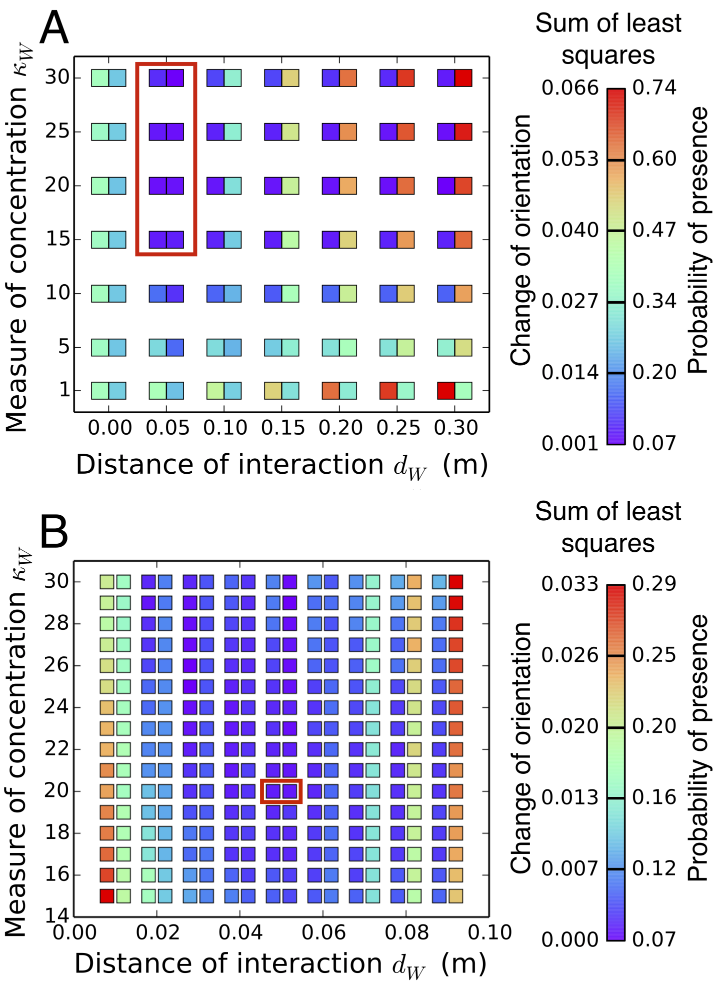

Figure 3: Fitting by sum of least squares minimisation of the parameters d_w and κ_W determining the interaction of a single fish with the walls of the tank. The parameter d_w corresponds to the threshold distance of interaction with the walls and κ_W is inversely proportional to the width of the von Mises distribution associated with wall following. To each couple (d_w, κ_W) corresponds a couple of squares whose colours indicate the value of the sum of least squares obtained for the comparison of the changes of direction (left square) and the probability of presence (right square) with the experimental data. Lower values of sum of least squares corresponding to better fits are in the purple and blue colour scale. (A) The exploration of a first set of parameter values indicated that the best couple (d_w and κ_W) are found for values ($d_w = 0.05$ and $15 < \kappa_W < 30$), highlighted in the red rectangle. (B) The refinement of the parameters exploration in the ranges determined in (A) showed that the best fitting is obtained by $d_w = 0.05$ and $\kappa_W = 20$.

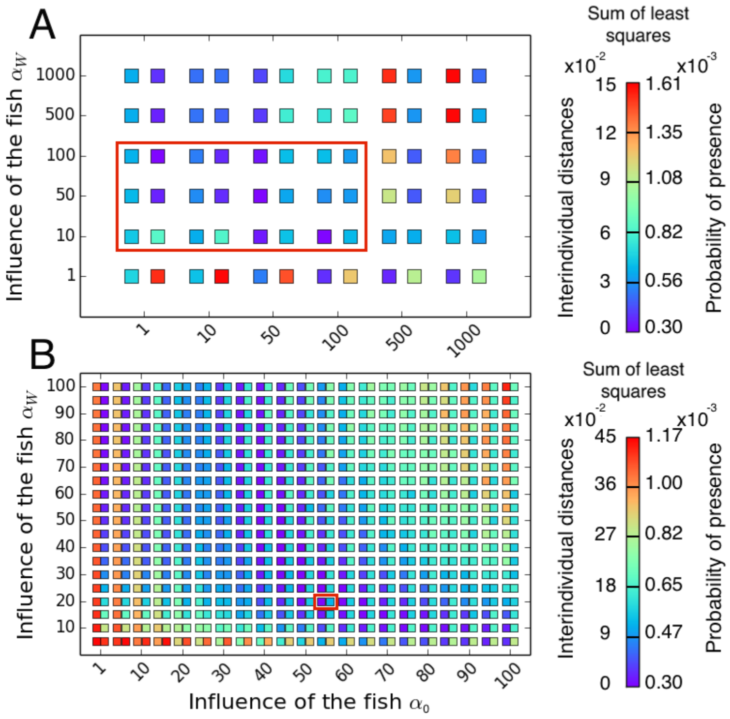

Figure 4: Fitting by sum of least squares minimisation of the parameters α_0 and α_W determining the interaction of a focal fish that far away or close to a wall with other individuals. To each couple (α_0, α_W) corresponds a couple of rectangles whose colours indicate the value of the sum of least squares obtained for the comparison of the interindividual distances (left rectangle) and the probability of presence (right rectangle) with the experimental data. Lower values of sum of least squares corresponding to better fits are in the purple and blue colour scale. (A) The exploration of a first set of parameter values indicated that the best couples (α_0, α_W) are found for values ($\alpha_0 \leq 100$, $10 \leq \alpha_W \leq 100$), highlighted in the red rectangle. (B) The refinement of the parameters exploration in the ranges determined in (A) showed that the best fitting is obtained by $\alpha_0 = 55$ and $\alpha_W = 20$.

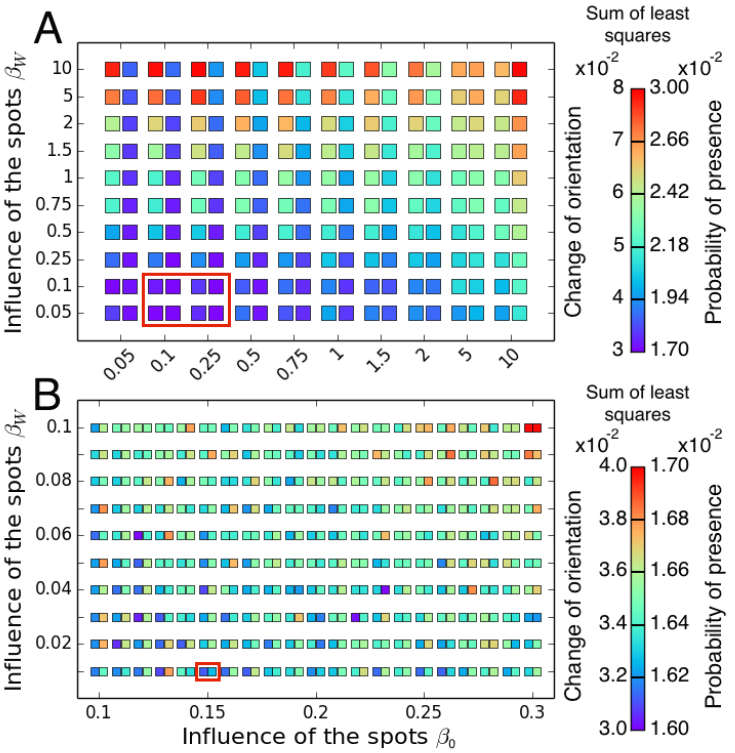

Figure 5: Fitting by sum of least squares minimisation of the parameters β_0 and β_W determining the interaction of a focal fish that far away or close to a wall with the spots of interest. To each couple (β_0, β_W) corresponds a couple of rectangles whose colours indicate the value of the sum of least squares obtained for the changes of orientation (left square) and the probability of presence (right square) with the experimental data. Lower values of sum of least squares corresponding to better fits are in the purple and blue colour scale. (A) The exploration of a first set of parameter values indicated that the best couples (β_0, β_W) are found for values ($0.05 \leq \beta_0 \leq 0.5$, $0 \leq \beta_W \leq 0.25$), highlighted in the red rectangle. (B) The refinement of the parameters exploration in the ranges determined in (A) showed that the best fitting is obtained by $\beta_0 = 0.15$ and $\beta_W = 0.01$.

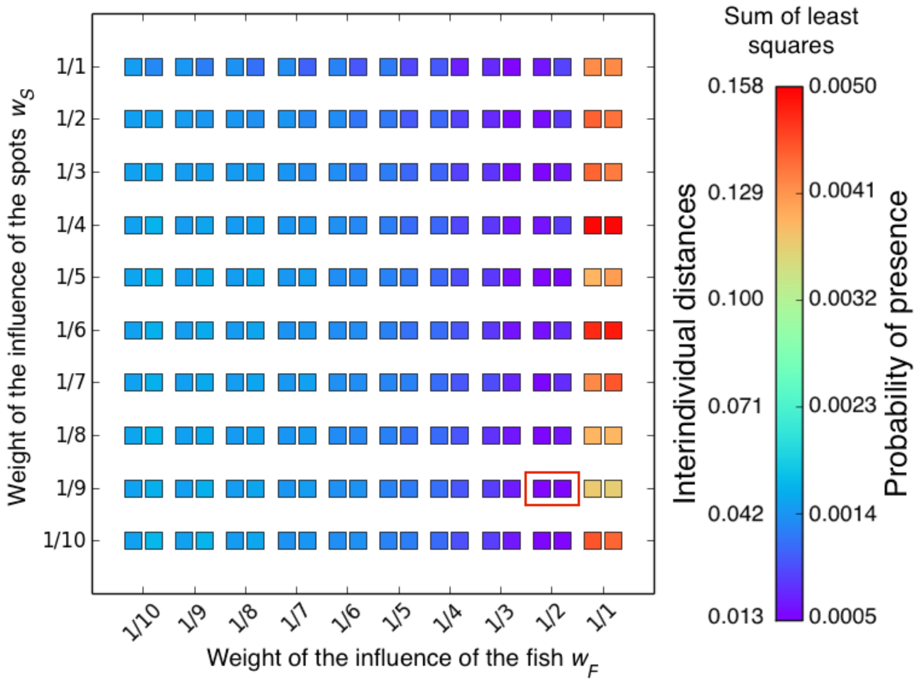

Figure 6: Fitting by sum of least squares minimisation of the parameters weighting the influence of the fish w_F and the spots w_S in simulation with 10 zebrafish swimming with two spots of interest. To each couple $(w_F,\, w_S)$ corresponds a couple of rectangles whose colours indicate the value of the sum of least squares obtained for the comparison of the interindividual distances (left square) and the probability of presence (right square) with the experimental data. Lower values of sum of least squares corresponding to better fits are in the purple and blue colour scale. The exploration of a set of parameter values indicated that the best couples $(w_F,\, w_S)$ is $w_F = \frac{1}{2}$, $w_S = \frac{1}{9}$.
